# Diagnostic work-up in patients with possible asthma referred to a university hospital

**DOI:** 10.3402/ecrj.v2.27768

**Published:** 2015-07-07

**Authors:** Vibeke Backer, Asger Sverrild, Charlotte Suppli Ulrik, Uffe Bødtger, Niels Seersholm, Celeste Porsbjerg

**Affiliations:** 1 Respiratory Research Unit, Department of Respiratory Medicine, Bispebjerg University Hospital, Copenhagen, Denmark; 2 Department of Pulmonary Medicine, Hvidovre University Hospital, Copenhagen, Denmark; 3 Department of Respiratory Medicine, Næstved Hospital, Næstved, Denmark; 4 Department of Respiratory Medicine Y, Gentofte University Hospital, Copenhagen, Denmark

**Keywords:** Asthma management, diagnostic tools, airway hyperresponsiveness, spirometry, reversibility, sensitivity, specificity

## Abstract

**Objective:**

The best strategy for diagnosing asthma remains unclear. Accordingly, the aim of this study was to evaluate diagnostic strategies in individuals with possible asthma referred to a respiratory outpatient clinic at a university hospital.

**Methods:**

All individuals with symptoms suggestive of asthma referred over 12 months underwent spirometry, bronchodilator reversibility test, Peak expiratory flow rate (PEF) registration, and bronchial challenge test with methacholine and mannitol on three separate days. The results of these tests were compared against an asthma diagnosis based on symptoms, presence of atopy and baseline spirometry made by a panel of three independent respiratory specialists.

**Results:**

Of the 190 individuals examined, 63% (*n*=122) were classified as having asthma. Reversibility to β_2_-agonist had the lowest sensitivity of 13%, whereas airway hyperresponsiveness to methacholine had the highest (69%). In contrast, specificity was the highest for reversibility testing (93%), whereas methacholine had the lowest specificity (57%). The combination of reversibility, peak-flow variability, and methacholine yielded a cumulative sensitivity of 78%, albeit a specificity of 41%. In comparison, a combination of reversibility and mannitol resulted in a specificity of 82% and a sensitivity of 42%.

**Conclusion:**

In this real-life population, different diagnostic test combinations were required to achieve a high specificity for diagnosing asthma and a high sensitivity, respectively: Our findings suggest that the diagnostic test approach should be based on whether the aim is to exclude asthma (high sensitivity required) or confirm a diagnosis of asthma (high specificity required).

Asthma is the most common chronic illness in adolescents and young adults living in western societies (1, 2). It is often under-diagnosed, which may relate to variability of the disease over time and between patients (3).

Asthma is characterized by respiratory symptoms combined with variable and reversible airflow obstruction. A diagnosis of asthma is objectively verified by the demonstration of variable airflow obstruction, either by reversibility to bronchodilators or steroids, spontaneous variation assessed by peak expiratory flow rate monitoring, or airway hyperresponsiveness (AHR) assessed by airway challenge tests such as exercise, methacholine, or mannitol (4). The diagnostic tools in asthma are numerous. Although asthma can be diagnosed solely based on relevant symptoms, in questionable cases, the diagnosis should be confirmed by an objective measure.

Diagnostic tests are characterized by the sensitivity and specificity. One test rarely performs well on both parameters but has either a high sensitivity (and the ability to rule out disease in case of a negative test) or a high specificity (and the ability to rule in disease in case of a positive test) (5). An overlap between diagnostic tests for asthma exists, but with a significant variation between individuals, probably due to the heterogeneous nature of what is currently identified or classified as ‘asthma’. It is well known that reversibility to β_2_-agonists has a low sensitivity due to many patients having a normal or near-normal lung function (3, 6), and in these cases, bronchial challenge testing is recommended to confirm the presence of the disease. Bronchial challenge tests have been designed with a wide range of different stimuli, including exercise, hyperventilation, hypertonic saline, mannitol, methacholine, and histamine, and all have different diagnostic properties (5). However, the best combination of diagnostic tests for asthma, including bronchial challenge tests, in unselected asthma patients in a real-life situation, has yet to be demonstrated. The aim of the present study was to evaluate the different diagnostic tools and to determine the best strategy for applying these tests in a real-life unselected population of patients with suspected asthma in a specialist setting.

## Material and methods

### Design

**Fig. 1:**
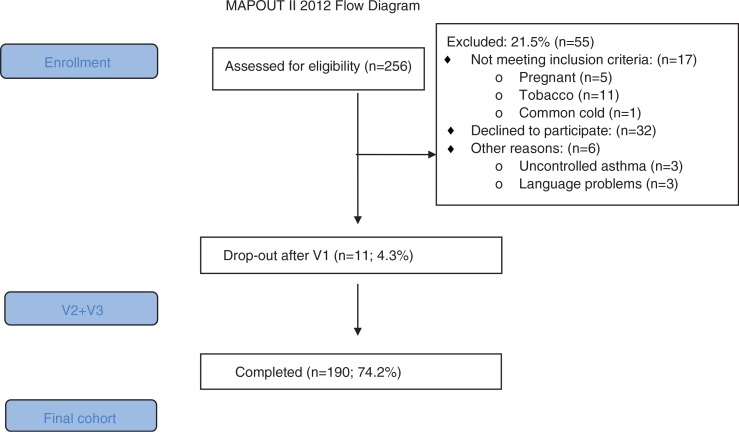
Flow-chart of the MapOut II study.

This is a cross-sectional study of individuals with possible asthma referred to the respiratory outpatient clinic at Bispebjerg University hospital, Copenhagen, Denmark. This group of patients is unselected and represent asthma patient referred to a university hospital. The participants were consecutively enrolled over 12 months (May 2012 to April 2013) (Fig. 1). Exclusion criteria were respiratory diseases other than asthma (e.g. sarcoidosis, chronic obstructive pulmonary disease (COPD)), children younger than 15 years, individuals older than 40 years with a smoking history of more than 10 pack-years, pregnancy, and recent respiratory infection (<6 weeks). All participants were assessed with a 3-day asthma evaluation program (Table 1): (V1) interview and reversibility test; (V2) methacholine provocation test, skin prick test, and asthma control questionnaire (ACQ); (V3) fractional exhaled nitric oxide (FeNO), mannitol provocation, and peak expiratory flow (PEF) diary. A specialist panel evaluated the diagnosis of asthma based on symptoms, family history of atopy, and baseline lung function. The specialist diagnosis of asthma was used to evaluate the diagnostic value of reversibility to β_2_-agonist (>200 mL and 12%), PEF variation (>20%), and AHR to methacholine (PD20<7.8 µmol) or AHR to mannitol (PD15<635 mg). The study was approved by the local ethics committee (H-3-2011-121).

**Table 1:** The distribution of tests performed on the three separate visits within 2–3 weeks

Visit 1 (V1)	Visit 2 (V2)	Visit 3 (V3)
Informed consent	*Methacholine*	*Collection of PEF cards*
Inclusion/exclusion	Skin prick test	*Mannitol*
Weight/Height	Questionnaire	
*FEV1 with reversibility*		
*PEF instruction*		

Tests included in the analysis of the diagnostic value of asthma tests are written in italic.

#### Study population

A total of 256 patients with possible asthma (age 15 years or more) were referred to the department during the 12-month study period from 1 May 2012 to 30 April 2013 (Fig. 1).

#### Evaluation algorithm

Height was measured in centimeters without shoes; weight was measured in kilograms with the participant wearing light clothing. BMI was calculated as weight in kg/height in m^2^. Before all study visits, all participants were requestedto refrain from short- and long-acting β_2_-agonists for 12 h, leukotriene antagonist for 24 h, and theophylline or antihistamine for at least 24 h. Intensive exercise was not allowed 6 h prior to mannitol testing, and furthermore patients were asked to abstain from inhaled corticosteroid (ICS) morning doses on the day of testing. In the case of an FEV_1_<70%, challenges with mannitol and methacholine were not performed.

### Visit V1

#### Spirometry

Lung function was measured using maximum expiratory flow volume according to the standards specified by the ERS and ATS (7), using EasyOne (Spiropharma^®^). Predicted normal values were calculated using NHANES reference values (7), and percentages of predicted normal values were then estimated. A reversibility test was performed, using four puffs of terbutaline. A significant reversibility was defined as a 12% increase in FEV1 (and minimum 200 mL).

### Visit V2

#### Questionnaire

Severity of asthma was classified according to the GINA guidelines as well-controlled, partly controlled or uncontrolled (8, 9). Severity of symptoms was classified according to the ACQ6 Juniper questionnaire (10), and asthma quality of life was measured by a standard questionnaire (miniAQLQ) (11). Tobacco consumption was recorded; the average number of pack-years was calculated for both smokers and ex-smokers [(average number of cigarettes*years)/20].

#### Skin prick test

Skin prick test was performed according to European standards with a panel of 10 standard allergen extracts (12). A cutoff value of 3 mm defined atopy.

#### Methacholine bronchial provocation

The method of measuring airway responsiveness toward inhaled methacholine has been described by Crapo et al. (13). A Jaeger nebulizer generated the aerosols (Jaeger GmbH, Germany) starting with a dose of isotonic saline followed by increasing cumulated doses of methacholine, ranging from 0.06 to 7.8 mol. Participants with an FEV1 reduction of 20% within a cumulative dose of methacholine of ≤7.8 mol have a positive test (PD20, i.e. AHR).

### Visit V3

#### Mannitol bronchial provocation

The participants inhaled an empty capsule followed by capsules with increasing doses of mannitol (from 5 to 635 mg) until maximum dose was reached or a 15% reduction in FEV_1_ (PD15) was achieved (AHR, i.e. a PD15, <635 mg) (14).

#### Flow variability (PEF)

All participants were instructed at Visit 1 (V1) to measure PEF twice daily for 2 weeks and collection of the PEF diary cards was done at Visit 3 (V3). A significant variability was defined as a day-to-day variation of at least 20% [(maximum – minimum)/maximum] (15).

#### Definition of asthma

The three independent specialists in respiratory medicine (CSU, UB, and NS), employed at hospitals other than Bispebjerg hospital, were e-mailed a file containing information on respiratory symptoms, family history of atopy and baseline spirometry (%pred), but not reversibility testing. Based on these data, each specialist independently decided whether each individual was ‘very likely to have asthma’, ‘likely to have asthma’, ‘unlikely to have asthma’, or ‘very unlikely to have asthma’. When two out of three scored an individual as ‘very likely’ or ‘likely’ to have asthma, the individual was classified as having asthma. In the event of two of the three specialists scoring an individual as ‘unlikely’ or ‘very unlikely’ to have asthma, the individual was classified as not having asthma.

### Statistics

The data were analyzed with the statistical software SPSS version 20.0 (Chicago, Illinois). Mean and standard deviations (±SD) were calculated for the normally distributed data. The chi-squared test was used when categorical variables was used. For the continuous variables, data were analyzed by ANOVA followed by the two-sample *t*-test to compare the groups. Sensitivity, specificity, positive predictive values (PPV), and negative predictive values (NPV) were calculated. Thereafter, the 95% confidence interval (95% CI) was calculated for each value. The cumulated sensitivity and specificity of the four different asthma tests were measured. When describing the different strategies, the selection of the test order was made from a clinician's point of view when selecting seven of the 16 possible combinations. The test most often applied in the clinical setting, the reversibility test, was selected as the first of the many test strategies. Values of *p*<0.05 were considered significant.

## Results

Of the190 participants included, the specialist panel classified 122 (64%) as having asthma. Of those referred, 150 had already been prescribed β_2_-agonist, of whom 45 were later classified as non-asthmatic. All three specialists suggested the same classification in 42% of the participants. Specialists S1 and S2 had a 65% [kappa coefficient (κ=0.3)] overlap in their evaluations, and specialists S1 and S3 had a 64% (κ=0.2) overlap; specialists 2 and 3 had a 54% overlap (κ=0.1).

Those defined as having asthma were characterized by more frequently being atopic, having not well-controlled asthma, as assessed by ACQ (ACQ>1.5: 48% vs. 18%, *p*<0.001), and low quality of life score (miniAQLQ: 5.5 vs. 6.1, *p*<0.001, Table 2) compared with those without asthma. On the other hand, no significant differences were found between the two groups in baseline level of lung function, reversibility to inhaled β_2_-agonist (12%) or PEF day-to-day variation (20%) (Table 2). However, those who were classified as having asthma more often had AHR to both methacholine (69% vs. 47%, respectively; *p*<0.01) and to mannitol (PD_15_ 36% vs. 16%, respectively; *p*<0.01) compared to those without asthma (Table 2). Lastly, those classified as having asthma had more often been prescribed anti-asthma therapy prior to referral (Table 3).

**Table 2:** Baseline characteristics of the 190 patients with asthma-like symptoms enrolled in the present study

	Non-specific respiratory symptoms (*n*=68)	Asthma (*n*=122)	Total (*n*=190)	*p*
	68 (36%)	122 (64%)	190 (100%)	
% females	57	57	57	NS
% current hay fever	41	59	52	<0.05
Age [mean (SD)]	32.5 (13)	31.9 (13)	32.1 (13)	NS
BMI	23.4 (3)	24.2 (5)	23.9 (4)	NS
FEV1 L	3.8 (0.9)	3.7 (0.9)	3.8 (0.9)	NS
FEV1 % predicted	97 (17)	95 (17)	95 (17)	NS
% FEV1/FVC ratio	83 (9)	81 (10)	81 (10)	NS
% with atopy	44	63	56	<0.01
% with AHR methacholine	47	65	62	<0.01
% with AHR mannitol	16	38	29	<0.01
% PEF cutoff>20%	42	39	40	NS
% reversibility cutoff>12%	8	13	11	NS
ACQ	0.9 (0.8)	1.6 (1.0)	1.3 (1.0)	<0.001
% with ACQ>1.5	18	48	37	<0.001
MiniAQLQ	6.1 (0.8)	5.5 (1.0)	5.7 (1.0)	<0.001

Mean (SD) analyzed with *t*-test and percentage of column analyzed with *X* ^2^-test.

**Table 3:** Baseline anti-asthma treatment of the 190 patients with asthma-like symptoms enrolled in the present study

	Non-specific respiratory symptoms	Asthma	Total	*p*
	68 (36%)	122 (64%)	190 (100%)	
% ICS treated^a^	22	38	33	0.058
% ICS/LABA treated^a^	20	35	30	0.057
% β_2_ treated^b^	66	86	79	0.01

a Patients with a history of inhaled steroid (ICS) with and without long-acting β_2_-agonist (LABA)

b Patients with a history of bronchodilator treatment.

As shown in Table 4, reversibility testing had the lowest sensitivity for a diagnosis of asthma but the highest specificity (11 and 95%, respectively), whereas methacholine challenge had the highest sensitivity albeit together with a low specificity (65 and 57%, respectively).

**Table 4:** Sensitivity and specificity (including CI 95% in brackets), as well as positive predictive value of a positive test (PPV) and a negative test (NPV) of selected variable of importance for the diagnosis of asthma

	Sensitivity	Specificity	PPV	NPV
β2 reversibility>12%	13 (8–15)	93 (85–97)	75 (52–90)	36 (33–38)
PEF variation>20%	39 (32–46)	58 (48–69)	59 (48–69)	39 (31–46)
Mannitol^a^	38 (34–44)	82 (71–89)	79 (69–88)	42 (37–46)
Methacholine^b^	65 (64–75)	57 (43–64)	74 (68–80)	48 (38–57)

a *p*=0.015

b *p*<0.01.

The outcome of the different combined test strategies was analyzed, and the sensitivity and specificity of seven test combinations are summarized in Table 5. When applying all four tests, the cumulative sensitivity reached 81%, albeit at the cost of a low specificity (41%). When analyzing the combination with the highest specificity, reversibility testing and the mannitol test resulted in a cumulative specificity of 75%, with a sensitivity of 42%. Addition of peak-flow monitoring increased the sensitivity to 54% but simultaneously decreased the specificity to 54%.

**Table 5:** Sensitivity and specificity of different combinations of diagnostic tests

	Asthma test	Test 1 (%)	Test 2 (%)	Test 3 (%)	Test 4 (%)	Test 5 (%)	Cummulative percent sensitivity	Cummulative percent specificity
Test	Reversibility >12%	11					11	93
Strategy 1	AHR mannitol (PD15)		32				42	75
	PEF variation >20%			12			54	54
	AHR methacholine (PD20)				26		80	41
	Negative results					20	–	–
Test	Reversibility>12%	11					11	93
Strategy 2	PEF variation>20%		21				32	63
	AHR mannitol (PD15)			22			54	54
	AHR methacholine (PD20)				26		80	41
	Negative results					20	–	–
Test	Reversibility>12%	11					11	93
Strategy 3	AHR methacholine (PD20)		46				57	54
	PEF variation>20%			21			78	41
	AHR mannitol (PD15)				3		80	41
	Negative results					20	–	–
Test	Reversibility >12%	11					11	93
Strategy 4	AHR mannitol (PD15)		32				42	75
	PEF variation >20%			12			54	54
	AHR methacholine (PD20)				26		80	41
	Negative results					20	–	–
Test	AHR to methacholine (PD20)	65					65	57
Strategy 5	PEF variation>20%		1				66	42
	AHR to mannitol (PD15)			7			73	42
	Reversibility>12%				7		80	40
	Negative results					20	–	–
Test	AHR to mannitol (PD15)	38					38	82
Strategy 6	AHR to methacholine (PD20)		35				73	56
	PEF variation>20%			7			79	43
	Reversibility>12%				1		80	41
	Negative results					20	–	–
Test	AHR to methacholine (PD20)	65					65	57
Strategy 7	AHR to mannitol (PD15)		7				72	56
	PEF variation>20%			7			79	43
	Reversibility>12%				1		80	41
	Negative results					20	–	–

Lastly, in a real-life situation, patients referred under the diagnosis possible asthma might be tested with metacholine first, “to rule out” those without asthma. A negative methacholine test was found in 50 (41%) patients classified as likely to have asthma according to the specialist panel, of whom 19 (17%) did not receive treatment with ICS, and should, therefore, probably be ruled out as having asthma. In comparison, of those classified as ‘asthma unlikely’ by the specialist panel, 39 (62%) had a negative methacholine test, of whom 27 (45%) were not treated with ICS. Due to a negative methacholine provocation, 46 (24%) patients were ruled out as asthma patients. With this approach, 42% was positive to methacholine only, further 20% were positive to mannitol, 24% had day-to-day variation with PEF, and 13% showed bronchodilator reversibility.

In this group of patients with newly diagnosed asthma having symptoms and a positive methacholine, the sensitivity of reversibility testing, mannitol testing, and PEF variation was 13, 37, and 41%, respectively, with a specificity of 98, 91, and 64%, respectively.

## Discussion

In this real-life asthma study examining unselected newly referred patients with potential asthma using a selection of asthma tests common in both asthma diagnosing and management, we showed that no single test alone was the optimal test to use but that the diagnostic accuracy increased with multiple testing. Overall, we showed that with a combination of diagnostic tests, 81% of asthma cases could be confirmed objectively in this population of patients referred to a specialist asthma clinic.

The entire analysis of the diagnostic validity of the different asthma tests has been both supported and questioned in a recent review analyzing the similarities and differences between BTS, NHBLI, and GINA guidelines (16). Diagnostic tests for asthma have well-recognized challenges, which include the practical feasibility of the tests, the mismatch of sensitivity, and specificity of each test, but also to the lack of a gold standard for the diagnosis of asthma, which makes validation of diagnostic tests difficult. The major drawback is the diversity of the asthma phenotypes combined with the fact that many patients with asthma symptoms also have other illnesses. Asthma-like symptoms are frequently observed in other non-asthmatic illnesses such as the early stages of COPD (17), laryngeal obstruction (18, 19), gastroesophageal reflux (19), poor physical fitness (20), sports-related shortness of breath or cough (21), obese (22), and cardiac diseases (23), and objective measurements are therefore of significant importance and necessary to eliminate misclassification.

The different asthma tests used in the present paper are in accordance with the recommendations of the different guidelines (10, 16), including the newly revised GINA guidelines (24). We showed that these commonly used tests, such as reversibility to β_2_-agonist and peak-flow day-to-day variation, had limited usefulness in this tertiary setting at a university hospital. The reversibility test had low sensitivity, whereas the peak-flow variation showed a better sensitivity but very low specificity for diagnosing asthma (24). This was not substantially changed by selecting those patients who had low lung function (13% vs. 20%, data not shown); notably, it was not changed by eliminating those treated with inhaled steroid at the time of referral – in contrast, those patients had a higher response than those who were untreated (17% vs. 8%, data not shown). This suggests that patients who are prescribed ICS by their GP are the ones who have more severe asthma, e.g. a confounding effect, where those who have significant AHR have more symptoms and hence are more likely to be prescribed ICS.

The present survey does not support the recommendation of starting with reversibility testing before any challenge testing, whereas the use of direct and indirect challenge tests was found to have a better distribution of sensitivity and specificity; methacholine had the highest sensitivity for detection of asthma (Table 4), but mannitol had the best association between sensitivity and specificity (Table 4). These findings are somewhat different from earlier findings in selected groups of asthma patients (25) and random population studies (26, 27). In an unselected group of young adults, the sensitivity of methacholine and mannitol was found to be 80% for both (27), which is substantially higher than in the present study where the sensitivity to methacholine was 65% and to mannitol 38%.

When applying all four diagnostic tests, we had a success rate of 81% for detecting asthma. When starting with a bronchial challenge test, the sensitivity increased more rapidly than when using standard tests such as the reversibility test and PEF variation (Table 5). A combined sensitivity of 81% is relatively satisfactory in this real-life asthma study performed in a hospital setting where some patients had already been prescribed anti-asthma therapy and others had not. When aiming for a workable solution in the everyday clinical setting, this study showed that such a solution should include either mannitol or methacholine, depending on the clinical setting. However, the specificity for mannitol was substantially higher than for testing with methacholine.

Although by their nature clinical guidelines are general recommendations aimed toward implementation in the clinical setting, they are generally based on the evidence from studies of highly selected patient populations (24). Accordingly, it is important that guideline recommendations are also tested in real-life studies, such as the present study and studies by others (28). When managing patients with typical asthma-like symptoms, as in the GP setting, guidelines might be focusing on symptoms, whereas those in a hospital setting might be more diverse, and a symptoms-based diagnosis is most likely inadequate (29). In a specialist setting, where the heterogeneity of asthma, with co-morbidities and different asthma phenotypes, is more the rule than the exception, these patients are more difficult to diagnose, treat and monitor. The correct diagnosis of asthma is paramount in achieving satisfactory asthma management with treatment and to secure reduced levels of sickness, unemployment, and early retirement (30, 31).

With another approach, where all patients with asthma-like symptoms and a negative provocation to methacholine were ruled out as not having asthma, showed similar findings toward testing with mannitol, PEF, and reversibility testing, among those with certain asthma selected by the specialist panel. High specificity was found with this approach, even higher than found with the specialist selection.

A potential limitation of the present study is the diagnosis of asthma. We used a specialist panel to classify the patients as having asthma or not having asthma. Despite the members of the asthma panel all having extensive clinical and scientific experience, the overlap between their classifications was low, although the kappa values were fair. However, under the given circumstances with no gold standard, we believe this was the most unbiased evaluation of disease taking the aim of evaluation of diagnostics tests into account. Another limitation might have been the wash-out period, which at least in patients treated with ICS, might have been too short. On the other hand, our results would be similar to findings among other out-patients clinics. Lastly, although no cost analysis have been performed, asthma testing is costly if all four challenge tests are needed; on the other hand, asthma is a chronic disease, and the diagnosis such be confirmed objectively to avoid risk of overtreatment.

In conclusion, in this real-life population, different diagnostic test combinations were required to achieve a high specificity for diagnosing asthma, and a high sensitivity, respectively. Our findings suggest that the diagnostic test approach should be based on whether the aim is to exclude asthma (high sensitivity required) or confirm a diagnosis of asthma (high specificity required).
